# Rezension der Ausstellung: *Normal#Verrückt – Zeitgeschichte einer erodierenden Differenz *(Heidelberg, Museum Sammlung Prinzhorn, 18.05.–28.09.2025)

**DOI:** 10.1007/s00115-025-01930-7

**Published:** 2025-12-10

**Authors:** Michaela Clark, Luisa Rittershaus

**Affiliations:** Institut für Geschichte, Theorie und Ethik der Medizin, Heinrich-Heine-Universität, Düsseldorf, Deutschland

Die von Thomas Röske und Maike Rotzoll kuratierte Ausstellung präsentiert die Ergebnisse eines von der DFG (Deutsche Forschungsgemeinschaft) geförderten Forschungsprojekts (2021–2024) zu der sich wandelnden Grenze zwischen „normal“ und „verrückt“ in Psychiatrie und Gesellschaft des Nachkriegsdeutschlands. Durch die präzise Auswahl und Anordnung von Artefakten eröffnet sie Einblicke in die Konstruktion, Infragestellung und Neubestimmung dieser Dichotomie im Laufe der zweiten Hälfte des 20. Jahrhunderts.

Die Ausstellung beginnt mit einer symbolischen Geste: Die beiden Worte des Titels „normal“ und „verrückt“ werden als Lichtinstallation auf den Boden projiziert und formulieren auf subtile Weise schon die Kernthese der Ausstellung. Beim Durchschreiten der Projektion verzerren sich die Wörter auf den Körpern der Besucher:innen und vollziehen zudem eine Etikettierung. Die Besucher:innen können sich als „normal“ oder „verrückt“ labeln, ein subtiler Hinweis darauf, dass solche Zuordnungen auch Resultat historischer und gesellschaftlicher Zuschreibungen sind. Die Instabilität und Durchlässigkeit psychiatrischer und gesellschaftlicher Normen im Zusammenhang mit der psychischen Gesundheit werden augenscheinlich.

**Abb. 1:**
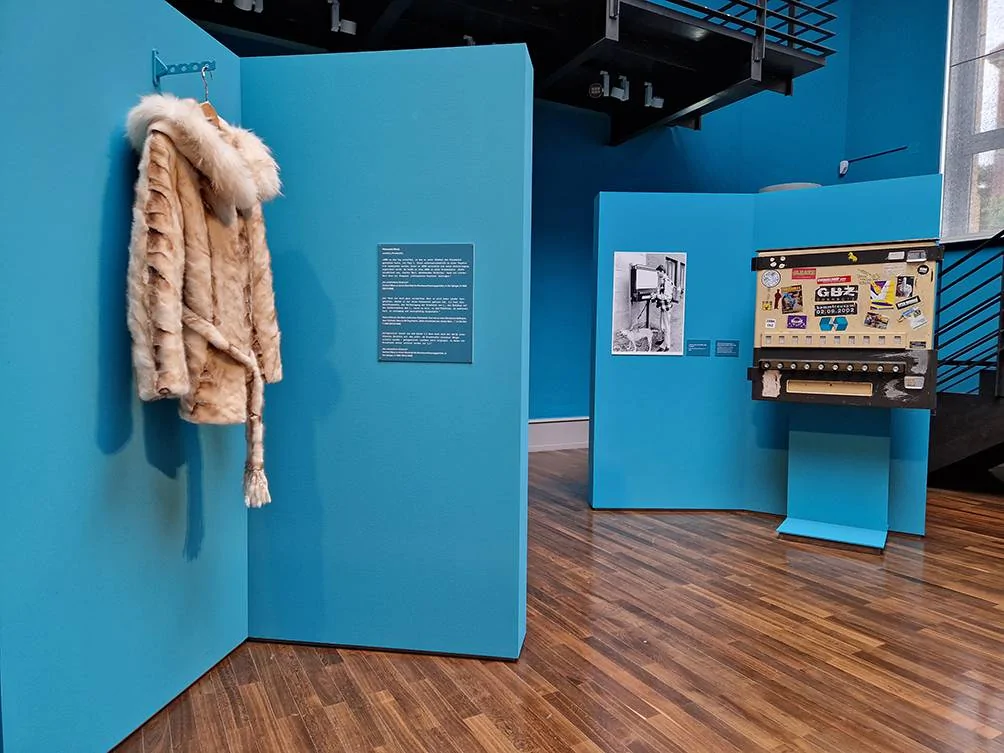
Installationsansicht im Erdgeschoss: Pelzmantel mit Bezug auf den Fall von „Paul L. Stein“ und Spritzenautomat

**Abb. 2:**
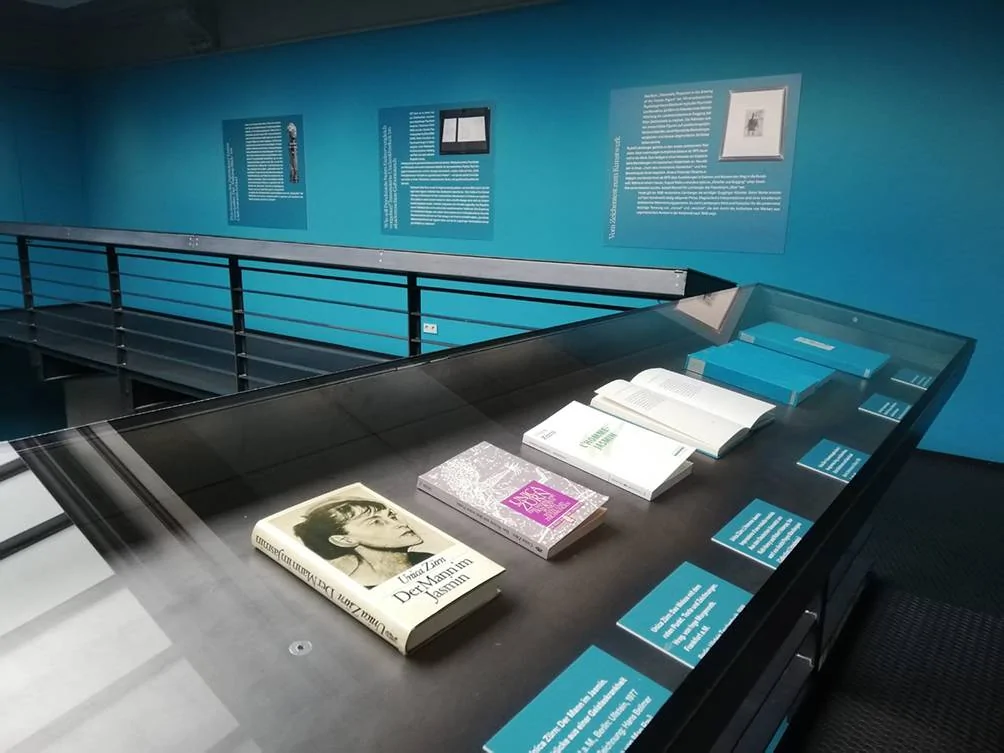
Installationsansicht auf der Empore

Die Installation der Ausstellung wurde sorgfältig inszeniert. Auf zwei Raumebenen entstehen unterschiedliche Wahrnehmungsmomente. Im Erdgeschoss präsentieren neun Displays jeweils Artefakte (Objekte, Filme, Dokumente oder Kunstwerke), die als Synonym für einzelne Forschungsstränge stehen (Abb. 1). Diese räumliche Struktur ermöglicht eine konzentrierte, fast intime Begegnung mit jedem Stück, bedeutet aber auch Fragmentierung und Isolation. Unmittelbar bei den Objekten fehlen erklärende Kontexte – ein verunsichernder, aber programmatischer Verzicht: Er fordert die Besucher:innen dazu auf, eigene Bezüge herzustellen und Neugier zu entwickeln. Auf der Empore wird diese Spannung aufgelöst: In Vitrinen finden sich weiterführende Publikationen und Archivmaterial, ergänzt durch erläuternde Tafeln (Abb. 2). Die im Erdgeschoss gezeigten Stücke erweisen sich als Schlüsselobjekte, die gemeinsam ein komplexes Narrativ zur Psychiatriegeschichte entwerfen.

Ein auffälliges Exponat ist ein Pelzmantel, der stellvertretend für die Geschichte von „Paul L. Stein“ steht. 1970 wegen des Diebstahls eines ähnlichen Stücks inhaftiert, wurde seine ursprünglich neunmonatige Haftstrafe durch Maßregelvollzug ersetzt – eine forensische Anordnung zur Zwangseinweisung von als psychisch krank geltende Personen. Was verurteilten Personen mit psychiatrischer Diagnose Unterstützung bieten sollte, führte bei Stein zu 15 Jahren unfreiwilliger Inhaftierung. Eine Entlassung war nur bei nachgewiesener „Gewaltfreiheit“ und psychiatrischer Genesung möglich – eine medikalisierte Kategorisierung im Gewand therapeutischer Sorge. Nachdem Steins Schicksal in den 1980er-Jahren breite öffentliche Aufmerksamkeit erlangt hatte, rückte die Frage nach dem „gewalttätigen“ Verhalten psychisch erkrankter Menschen verstärkt in den Fokus.

Vom Strafansatz zu unterstützenden Strategien: Die Ausstellung zeigt einen „Spritzautomaten“ aus den 1990er-Jahren – einen zum Spritzenverkauf umgebauten Zigarettenautomaten. Er markiert einen Paradigmenwechsel in der Gesundheitspolitik während der HIV/AIDS-Krise. Anstatt Drogenkonsument:innen als Randfiguren oder Kriminelle zu betrachten, bot der Automat eine niederschwellige Möglichkeit, an saubere Spritzen zu gelangen und damit ein Minimum an gesundheitlicher Prävention. Zugleich dienen die Automaten als diskrete Schnittstelle zu Beratungsangeboten – ein Symbol für den gesellschaftlichen Wandel therapeutischer Haltungen.

An einem der Ausstellungspaneele ist ein kleiner Bildschirm mit zwei Kopfhörern installiert, auf dem die Fernsehproduktion *Ohne Glas und Maß* (1960) läuft. Entstanden als Beitrag zu einer öffentlichen Gesundheitskampagne, stammt der Film aus einer Zeit, in der Alkoholismus nicht mehr primär als individuelle moralische Schwäche, sondern zunehmend als soziale Krankheit begriffen wurde, der vorgebeugt oder die sogar geheilt werden konnte. Auslöser für diesen Diskurswandel war die Erkenntnis, dass immer mehr gesunde und vermeintlich „normale“ junge Menschen übermäßig Alkohol konsumierten.

Ein weiteres prägnantes Objekt ist der „Sprechstab“, ein zeremonielles Werkzeug des *Club Antonin Artaud* – eines Brüsseler Gruppentherapie-Kollektivs, 1962 von Patient:innen und Pflegekräften des Brüsseler Instituts für Psychiatrie gegründet. Das Touristenreplikat eines Tabwa-Artefakts wurde seit den 2000er-Jahren vom Club-Psychiater eingesetzt, um während der Diskussionsrunde eine teilnehmerzentrierte und respektvolle Gesprächskultur zu fördern. Die Mitglieder (statt „Patient:innen“) sprachen nur mit dem Stab in der Hand. Damit knüpfte man an frühere Ideale alternativer Therapien an – Kreativität und freie Ausdrucksformen, flache Hierarchien und eine auf Autonomie zielende Praxis.

Der kurze Briefwechsel zwischen dem Psychiater Hubertus Tellenbach (1914–1994) und dem Psychoanalytiker Paul Parin (1916–2009) aus dem Jahr 1971 steht als Synonym für den Konflikt der aufkommenden transkulturellen Psychiatrie in Bezug auf den Kulturvergleich bei psychiatrischen Erkrankungen. Die in diesen Schreiben verdichtete Spannung verweist auf mehr als nur eine fachinterne Debatte: Sie spiegelt eine Neubewertung eurozentrischer medizinischer Paradigmen im Zuge der globalen Dekolonisation wider und öffnet den Blick auf einen tiefen theoretischen Riss im sich entwickelnden Bereich der Ethnopsychiatrie.

Mit einer gerahmten Zeichnung von Rudolf Liemberger (1937–1988), einem ehemaligen Patienten der psychiatrischen Klinik Maria Gugging in Österreich, thematisiert die Ausstellung die umstrittene Dichotomie zwischen psychiatrischer Pathologie und künstlerischer Kreativität. Liembergers Werk steht exemplarisch für die Neubewertung von einst anonymisiertem Klinikmaterial zu Kunst im Kontext der sog. „Art Brut“ in den 1970er-Jahren. Die Schöpfer:innen dieser Werke bewegten sich damit zwischen den Identitäten Patient:in und Künstler:in.

Ähnlich verweist das literarische Werk von Unica Zürn (1916–1970) auf die komplexen und auch hierarchischen Strukturen im Kontext von psychischer Erkrankung und künstlerischem Schaffen. Auch wenn Zürn für die surrealistische Ästhetik ihrer Kunst und ihres Schreibens gefeiert wurde, zeigt die posthume Veröffentlichung ihres Werks *Der Mann im Jasmin* (1977) ihre Texte als Produkt ihrer psychischen Erkrankung. Viele Texte entstanden zwar in einem Zustand seelischer Belastung, doch anstatt ihre literarische Eigenständigkeit zu betonen, rahmt der Verlag Zürns künstlerische Arbeit mit ihrer Geschlechtsidentität und psychiatrischen Diagnose.

Ein Exponat zum Anfassen ist das auf einem Sockel präsentierte „Wutkissen“. Doch was auf den ersten Blick als ein augenzwinkernder Konsumartikel erscheint, birgt Spuren gesellschaftlicher und politischer Diskurse. Es knüpft an die Praktiken feministischer Therapiegruppen der 1970er-Jahre an, in denen ähnliche Objekte als Ventil für den Ausdruck unterdrückter Gefühle dienten. Indem Frauen ermutigte wurden, ihren Zorn körperlich und stimmlich zu artikulieren sowie Wut als legitimen weiblichen Gefühlszustand zu etablieren, verkörperte dieses Werkzeug sowohl eine therapeutische Innovation als auch die politisierte Herausforderung patriarchalischer Normen.

Aufbauend auf diesem Thema des Persönlichen im Politischen steht ein vom Sozialistischen Patientenkollektiv (SPK) in Heidelberg verwendeter Matrizendrucker sinnbildlich für den Schnittpunkt zwischen politischem Widerstand und psychiatrischer Selbstbestimmung. Der Drucker wurde verwendet, um die selbstverfassten „Patienten-Infos“ des Kollektivs zu vervielfachen. Diese kollektive Akte des Sprechens, Schreibens und Druckens war nicht nur Ausdruck politischer Organisation, sondern Teil eines bewusst gestalteten therapeutischen Prozesses. Indem das SPK Kapitalismuskritik mit antifaschistischer Rhetorik, Satire und Selbstausdruck verband, schuf es ein eigenes Deutungsmodell, in dem der Kapitalismus als Ursache psychischen Leidens identifiziert wurde. Der Matrizendrucker zeugt von einer Bewegung, die Heilung und Revolution untrennbar dachte.

Der Ehrgeiz von *Normal#Verrückt* ist groß: Die Ausstellung versucht nicht nur, die unterschiedlichen Facetten eines multiinstitutionellen Projekts zu einem kohärenten Ganzen zu fügen, sondern auch akademische Forschungsergebnisse in eine visuell ansprechende und zugleich inhaltlich dichte Präsentation zu übersetzen. Eine Stärke liegt in der präzisen räumlichen Dramaturgie und den gezielten Objektimpulsen. Jedes Exponat wird historisch sowohl aus psychiatrischer als auch aus sozialer Perspektive kontextualisiert. Das sukzessive gespannte Netz aus Bezügen verlangt – auch durch die räumliche Trennung von Objekt und Metaebene – den Besucher:innen allerdings ein hohes Maß an Aufmerksamkeit ab. Ohne Vorkenntnisse zur Psychiatriegeschichte kann dies etwas überfordern. Hier werden Ausstellungskatalog sowie das Begleitprogramm zu einer ausgezeichneten Navigationshilfe.

*Normal#Verrückt* überzeugt sowohl als Ausstellung als auch als wissenschaftliche Intervention. Die Verbindung von historischem, medizinischem, politischem und künstlerischem Material eröffnet einen Zugang dazu, wie psychiatrische und gesellschaftliche Normen in Deutschland seit den 1950er-Jahren definiert, stabilisiert, verschoben und dekonstruiert wurden. Für Kliniker:innen bietet die Ausstellung den Anstoß zur kritischen Reflexion über epistemologische und ethische Grundlagen ihrer eigenen Praxis, für Historiker:innen ein Labor, in dem die vielen Fallstudien bestehende Narrative vertiefen. Und für die breite Öffentlichkeit schließlich ist sie eine Einladung – und eine Herausforderung – die eigenen Gewissheiten zu prüfen. Hier liegt eine zentrale Leistung der Ausstellung: Sie liefert keine direkten Antworten, sondern schärft den Blick auf die Kategorien, mit denen wir „normal“ und „verrückt“ überhaupt denken.

